# Welcome to the Journal of Cardiothoracic Surgery

**DOI:** 10.1186/1749-8090-1-1

**Published:** 2006-03-03

**Authors:** Vipin Zamvar, David P Taggart

**Affiliations:** 1 Department of Cardiothoracic Surgery, Royal Infirmary of Edinburgh, Edinburgh, UK; 2 Department of Cardiovascular Surgery, University of Oxford, Oxford, UK

## 

Journal of Cardiothoracic Surgery (JCTS) is an open access, peer-reviewed, online journal, which will encompass all aspects of research in the field of cardiothoracic surgery. JCTS will provide a forum for the publication and free-access of high quality scientific reports documenting clinical and experimental advances in cardiac and thoracic surgery, and related fields. The following types of articles will be accepted: Research Article, Review, Study Protocol, Book Review, Case Report, Case Study, Commentary, and Editorial. The intended audience is cardiothoracic surgeons, cardiothoracic anaesthesiologists, cardiologists, chest physicians, and allied health professionals.

There are many cardiothoracic journals in the printed form. Most of these are affiliated to national or international societies, and all are subscription-based, thus there is restricted access to the material published. The turnaround time for papers can also be quite long for some of these journals. JCTS will aim to provide a quick and efficient service to authors. Being open access, the material published will be freely available on the internet. All articles will be archived in major databases including PubMed Central.

The term open access is self-explanatory. In practical terms, it means that anybody with access to the internet will be able to access and download material published in the JCTS.

The main motivation for researchers and clinicians who write papers is the desire to advance the frontiers of knowledge in their speciality. What better medium than an open-access journal that allows anybody, anywhere unfettered access to the results of their work?

However, alongside this altruistic aim is a desire to be recognised for their work in a manner that will also help advance their careers. Citation counts of their papers, and impact factors of journals in which they publish are two objective (even though not entirely free of controversy) markers widely used in the scientific community. Open access journals will not put any author at a disadvantage. In fact there is some evidence that open access articles have a greater chance of being cited [1]. For articles published in the JCTS, authors will be able to view the number of times their article has been accessed. There is evidence that the number of accesses in the weeks after publication can be a potentially useful marker of subsequent citation count [2].

Authors also value open access, and consider this an important factor in deciding which journal to submit their work to [3].

The future of scientific publication is definitely headed in the direction of open access. The internet has profoundly changed the way we communicate and obtain information. For my nine-year-old daughter, Google is the first port of call when she has to do any research for her schoolwork. She has learnt that enough information is available free on the net, and she ignores sites that ask for a subscription to help her with her course work. In the years to come, she will continue to expect information to be freely available. In general, any information that is available freely (read open-access) is more likely to be looked at than information that is not (read subscription-based).

Open access publication, however requires funding. Authors of papers accepted for publication in JCTS will be asked to pay an article-processing charge (APC). The concept of authors contributing towards the cost of publication is not new. Many subscription-based journals including Circulation, Circulation Research, Neuron, Cell, Blood require authors to contribute towards the costs of publication.

Many research funding agencies strongly encourage open-access publication, and in fact allow the use of research grants for this purpose. These include the National Health Service, and the Wellcome Trust in the UK, and the National Institutes of Health in the USA [4]. Full or part waivers of the APC may be granted in cases of financial hardships and authors affiliated to BMC member institutes will also benefit from APC discounts.

Current methods of peer review are not perfect. A single paper can elicit two different opinions from two recognised experts. Even so, rigorous peer review remains the mainstay of quality control in scientific publishing. The JCTS has a distinguished international editorial board, which covers expertise in all areas of cardiac and thoracic surgery. Peer review will be rigorous, open and transparent. For all accepted papers the comments of the reviewers will be published as well. This is not only to give recognition to the peer reviewers for their work, but also to ensure accountability via openness and transparency. Reviewers as well as authors will be asked to declare any competing interests. Any reader will be able to follow the exchanges between the authors and reviewers before publication (something that is just not possible with paper based journals).

JCTS will also have a comment section, which will allow readers to comment on articles published in the journal. This is in effect post-publication peer review, and we would expect authors to respond to the comments in a timely manner.

With the launch of the JCTS we enter a new era of publishing in the field of cardiothoracic surgery. On behalf of the entire editorial board, David Taggart and I would like to welcome you to this exciting new journal.

## Competing interests

1) The majority of APC is used by BioMed Central to fund the tools required for peer-review and publication. VZ and DT are the editors-in-chief of JCTS and receive a proportion of the APC revenue from the journal.
